# Quiet Down Now: How Excitatory Neurons Inhibit One Another

**DOI:** 10.1371/journal.pbio.1000474

**Published:** 2010-09-07

**Authors:** Richard Robinson

**Affiliations:** Freelance Science Writer, Sherborn, Massachusetts, United States of America

**Figure pbio-1000474-g001:**
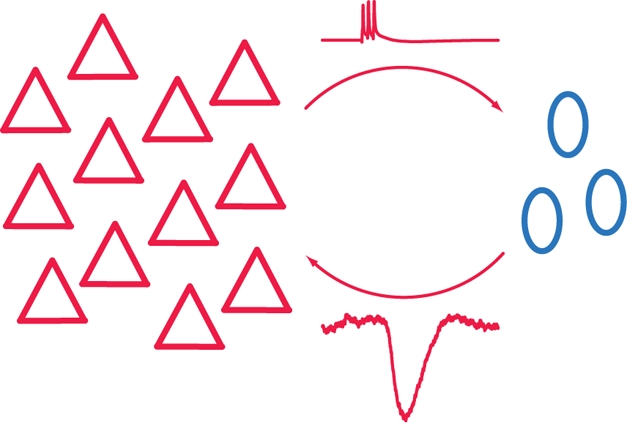
Bursts in the pyramidal cell population (red) trigger inhibition in the Martinotti cell population (blue), leading to self-inhibition of the pyramidal cell population.

[Fig pbio-1000474-g001]Draped atop the brain like a wrinkled gray blanket, the cortex is responsible for much of what we perceive, think, and do. It is a couple of square feet in area and only a few millimeters thick, but its millions of neurons form trillions of synaptic connections, making it, hands down, the most complex piece of neural real estate on the planet. Its macroscopic convolutions can only hint at the cortex's real intricacy, found at the microscopic level, where the thin cortical blanket is seen to be composed of millions of adjacent columns of cells.

Each column is less than a millimeter wide, is divided into distinct layers, and contains neurons numbering in the low thousands. Neurons within a column receive input from and send output to other areas in the brain, processing it in between. They do so by talking mainly to one another, rather than to neurons in adjacent columns, passing information from the bottom layer to the top, refining it along the way. These columns of cells are the functional units of the cortex.

Most of the neurons within a column, and therefore within the cortex as a whole, are so-called pyramidal neurons, whose output excites the cell it synapses with. Inhibitory cells, which damp down the activity of their target neurons, are far less numerous throughout the cortex. This imbalance gives rise to a puzzle—if excitatory neurons vastly outnumber inhibitory ones, how does the brain avoid runaway excitation? In this issue of *PLoS Biology*, Thomas Berger and colleagues provide one answer, showing that an increase in coordinated firing of a small number of pyramidal neurons within one layer of the column (layer 5, the fifth layer in from the cortical surface) activates a small number of nearby inhibitory neurons, leading ultimately to controlled, synchronized firing of the local pyramidal neuron population within the layer.

The authors built on recent work, including their own, demonstrating the importance of a type of inhibitory neuron called the Martinotti cell. In their previous studies, they showed that the Martinotti cell was critical for “frequency-dependent disynaptic inhibition.” Here, they characterized this important regulatory mechanism in detail, using microelectrodes to stimulate and record from multiple cells within layer 5 in the somatosensory cortex of the rat.

Pyramidal cells synapse onto other pyramidal cells, making a “monosynaptic” connection. They also synapse onto Martinotti cells, which in turn synapse with other pyramidal cells, making a “disynaptic” connection between the two pyramidal cells. The monosynaptic connection is excitatory, but, because the Martinotti cell makes GABAergic synapses onto its targets, the disynaptic connection is inhibitory. The authors found that as the firing frequency of the upstream pyramidal cell increased, the net effect on the downstream one went from excitatory to inhibitory, just the behavior one might want in a system to prevent runaway excitation. The downstream cells not only fired less, but when they did fire, they tended to fire in unison, so that the output of the column (layer 5 pyramidal neurons are the main output neurons of a column) as a whole became more synchronized.

A single upstream pyramidal cell synapsed onto a small handful of Martinotti cells, which in turn made connections to a large number of downstream pyramidal cells, providing a multiplier effect commonly seen in control systems. As more upstream cells were stimulated, the Martinotti cells fired faster, tightening the control on downstream cells. By varying the number of cells they stimulated, the authors found it took remarkably few pyramidal cells—four, to be precise—to inhibit the entire population of nearby pyramidal neurons, consisting of hundreds of neurons. Even brief synchronous bursts from three cells led to inhibition of large numbers of downstream cells.

The results obtained here apply strictly only to the particular neuronal types found in the single cortical layer studied, and it is open to question whether this mechanism is operating in other layers and in other regions of the brain. Nonetheless, the details uncovered in this study will be important for understanding how information flow within cortical columns is controlled, and ultimately how the column as a whole receives, computes, and releases information.

**Berger TK, Silberberg G, Perin R, Markram H (2010) Brief Bursts Self-Inhibit and Correlate the Pyramidal Network. doi:10.1371/journal.pbio.1000473**

